# A New Score for Predicting Acute Gastrointestinal Bleeding in Patients Administered Oral Antiplatelet Drugs

**DOI:** 10.3389/fphar.2020.571605

**Published:** 2021-01-14

**Authors:** Meina Lv, Xiaochun Zheng, Tingting Wu, Wenjun Chen, Shaojun Jiang, Hongqin Zhang, Fangda Xu, Jinhua Zhang

**Affiliations:** ^1^ Department of Pharmacy, Fujian Medical University Union Hospital, Fuzhou, China; ^2^ College of Pharmacy, Fujian Medical University, Fuzhou, China; ^3^ Department of Pharmacy, Fuzhou Second Hospital Affiliated to Xiamen University, Fuzhou, China; ^4^ Department of Information, Fujian Medical University Union Hospital, Fuzhou, China

**Keywords:** acute gastrointestinal bleeding, risk factor, score, aspirin, clopidogrel

## Abstract

Antiplatelet drugs may increase the risk of gastrointestinal bleeding. Currently, there is no specific score for predicting the risk of gastrointestinal bleeding caused by oral antiplatelet drugs. In this study, the gastrointestinal bleeding risk score was established and compared with the CRUSADE score in order to reduce the occurrence of clinical gastrointestinal bleeding events. Our study included 4052 patients who received oral antiplatelet drugs. Data were obtained from the patient medical records inpatient system. Cases of acute gastrointestinal bleeding and mortality were recorded. The bleeding score was established by logistic regression, area under the receiver operating characteristic curve, and the Hosmer–Lemeshow test. Finally, 171 patients had acute gastrointestinal bleeding. The mortality rates of patients in the bleeding and nonbleeding groups were 24.6 and 4.7%, respectively. A multivariate analysis revealed that an age of >65 years, anemia, recent major bleeding, a history of gastrointestinal bleeding, combined oral anticoagulants, and dual antiplatelet therapy are risk factors, and combined proton pump inhibitors are protective factors for acute gastrointestinal bleeding. We used these risk factors to establish a score for predicting acute gastrointestinal bleeding, named (ABC)_2_D score. The area under the curve for (ABC)_2_D score was 0.857 (*p* < 0.001), higher than the CRUSADE score of 0.693 (*p* < 0.001). The Hosmer–Lemeshow *p* value was 0.324. We developed the (ABC)_2_D score based on seven risk factors (i.e., age, anemia, recent major bleeding, a history of gastrointestinal bleeding, no-proton pump inhibitors use, combined oral anticoagulants, and dual antiplatelet therapy). (ABC)_2_D score was superior to the CRUSADE score. This new risk-scoring model may help to identify patients at a significant risk of gastrointestinal bleeding.

## Introduction

Antiplatelet drugs are the cornerstone of cardiovascular disease prevention and treatment worldwide (Sostres et al., 2019). Although antiplatelet drugs successfully reduce the risk of recurrent ischemic events, they increase the risk of severe bleeding (Antithrombotic Trialists’ Collaboration et al., 2009; Li et al., 2017), of which gastrointestinal bleeding is common (Yamada et al., 2012). Studies have shown that after treatment with antiplatelet drugs, during a 2.5-year follow-up, the incidence of acute gastrointestinal bleeding is approximately 11.9% (Pannach et al., 2017), the prognosis of patients who have experienced bleeding events is poor, and these patients are at an increased risk of mortality from cardiovascular disease. Patients with moderate or severe bleeding increased cardiovascular mortality to 2.05 times (HR 2.05, 95% CI 1.38–3.04) (Berger et al., 2011). Therefore, in order to prevent and reduce the risk of gastrointestinal bleeding, it is important to use risk models for acute prediction before patients receive antiplatelet drug treatment.

Given the widespread use of antiplatelet therapy and the serious consequences of bleeding events, it is important to predict the risk factors for gastrointestinal bleeding associated with antiplatelet drugs and to construct a risk score. Currently, there are several bleeding risk scores, such as the CRUSADE (Can Rapid Risk Stratification of Unstable Angina Patients Suppress Adverse Outcomes With Early Implementation of the American College of Cardiology/American Heart Association Guidelines) score (Subherwal et al., 2009) and the S_2_TOP-BLEED score (Hilkens et al., 2017). The main outcome of these scores is major bleeding, including intraocular hemorrhage, gastrointestinal bleeding, and intracranial hemorrhage. The severity of bleeding events may be affected by organs themselves or by the changes that occur in organs (Laine and Jensen, 2012; Strate and Gralnek, 2016), so the above models used to independently predict gastrointestinal bleeding may not be accurate. There is currently no specific score for predicting the risk of gastrointestinal bleeding caused by oral antiplatelet drugs.

In our study, patients administered oral antiplatelet drugs were followed up for 6 months to study the risk factors for antiplatelet drug-induced gastrointestinal bleeding and to establish a score to assess the risk of gastrointestinal bleeding. In addition, comparing our score with the CRUSADE score, which is currently recommended by Chinese experts to assess bleeding risk after antiplatelet therapy (Chinese Society of Cardiology of Chinese Medical Association and Editorial Board of Chinese Journal of Cardiology 2013), allows clinicians to better predict the risk of gastrointestinal bleeding, thereby optimizing antiplatelet therapy and reducing the incidence of gastrointestinal bleeding events.

## Methods

### Study Design and Population

**FIGURE 1:**
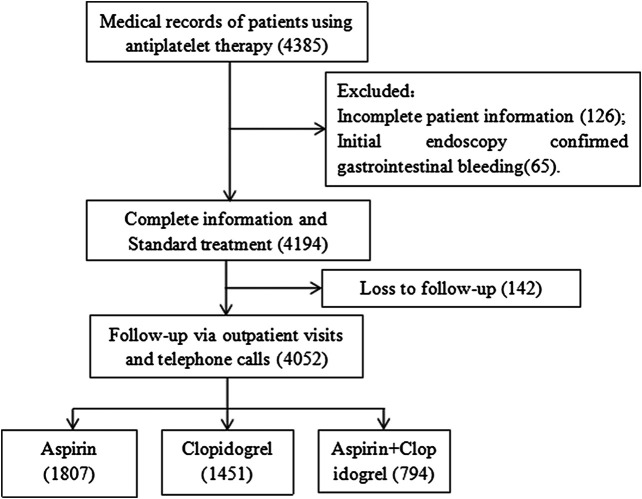
Flowchart of patient screening.

We retrospectively analyzed patients who used antiplatelet drugs for at least 6 months at two medical centers (Fujian Medical University Union Hospital (one of the largest emergency hospitals in China) and Fuzhou Second Hospital (affiliated to Xiamen University)) from May 2015 to December 2018. Data were obtained from the patient medical records inpatient system. We searched the electronic database using keywords such as “aspirin,” “clopidogrel,” and “antiplatelet drugs.” There were 4,385 patients taking antiplatelet drugs (aspirin 100 mg/day, clopidogrel 75 mg/day, or aspirin 100 mg/day plus clopidogrel 75 mg/day) continuously. The exclusion criteria were as follows: (1) age <18 years; (2) gastrointestinal bleeding before medication confirmed by endoscopy; (3) no follow-up or <6 months of follow-up; (4) insufficient data preventing subsequent analysis. Finally, 4,052 patients were included in the study (Figure 1). The study was approved by the Fujian Medical University Union Hospital Ethics Committee. Informed consent was obtained from all individual participants included in the study through telephone follow-up.

### Data Collection

We collected data on each patient from the electronic database at the hospital. Data were clinically organized and recorded by professional doctors and nurses in a timely manner, including demographic information (e.g., age and sex), concomitant illnesses (e.g., hypertension, diabetes, chronic kidney or liver disease, history of gastrointestinal bleeding, history of peptic ulcers, recent major bleeding (defined as major bleeding <30 days before starting antiplatelet drugs), and anemia (defined as a hemoglobin concentration of <13 g/dl in males or <12 g/dl in females)), and other medications (e.g., nonsteroidal anti-inflammatory drugs, proton pump inhibitors, and oral anticoagulants).

### Endpoints and Follow-Up

Patients were followed up at the antithrombotic clinic and by telephone for approximately 6 months. Patients were generally required to undergo regular outpatient follow-up every month to facilitate monitoring of symptoms and clinical signs. For patients with a bleeding tendency, further examination was recommended. The main outcomes were acute gastrointestinal bleeding and death. Gastrointestinal bleeding events were defined as bleeding with clinical evidence (i.e., guaiac-positive stool, melena, hematochezia, hematemesis, or the presence of blood in the gastrointestinal tract on endoscopic evaluation) and a documented decrease in hemoglobin of ≥2 g/dl (Chan et al., 2010; Yin et al., 2018). The cause of death was determined by consulting clinical information records and death certificates in the electronic medical records system.

### Statistical Analysis

SPSS 25.0 (IBM Corp., Armonk, NY, United States) statistical software was used to analyze data. Continuous variables are presented as mean ± standard deviation or median, and categorical variables are presented as percentages. Pearson’s *χ* ^2^ test or Fisher’s exact test was used for univariate analysis of categorical variables as appropriate. The variables related to acute gastrointestinal hemorrhage with a univariate analysis (*p* < 0.20) were included in the logistic regression analysis to confirm whether they were independent predictors of acute gastrointestinal hemorrhage. If the *p* value was <0.05, they were retained in the final model to establish a risk score. The weight of each predictor, according to the coefficients in the model, was determined. The area under the curve (AUC) was used to evaluate the discriminatory power of the risk-scoring model (bleeding group vs. nonbleeding group 1:1). The Hosmer–Lemeshow test was used to assess the calibration ability of the model. The logrank test and Kaplan–Meier curves were used to estimate the cumulative mortality of patients with gastrointestinal bleeding and patients without bleeding. A *p* value of ≤0.05 was considered statistically significant.

## Results

### Patient Characteristics

A total of 4,052 patients were included. Table 1 shows the baseline characteristics of patients taking antiplatelet drugs. The average age of patients in the bleeding group was greater than the average age of patients in the nonbleeding group (mean age, 72.6 vs. 64.1 years, respectively; *p* < 0.001). Certain factors, including an age of >65 years (76.0 vs. 48.0%, *p* < 0.001), male sex (74.3 vs. 65.5%, *p* = 0.019), drinking (31.0 vs. 20.5%, *p* = 0.001), recent major bleeding events (46.2 vs. 18.7%, *p* < 0.001), a history of gastrointestinal bleeding (36.8 vs. 1.2%, *p* < 0.001), a history of peptic ulcers (18.7 vs. 2.1%, *p* < 0.001), anemia (72.5 vs. 50.2%, *p* < 0.001), diabetes (35.1 vs. 21.8%, *p* < 0.001), hypertension (73.1 vs. 53.8%, *p* < 0.001), combined use of proton pump inhibitors (24.6 vs. 42.1%, *p* < 0.001), combined use of oral anticoagulants (36.3 vs. 4.1%, *p* < 0.001), and dual antiplatelet therapy (36.3 vs. 18.9%, *p* < 0.001) correlated with acute gastrointestinal bleeding (*p* < 0.05).

**TABLE 1:** Baseline clinical characteristics of patients.

Characteristics	GIB	No GIB	*p* value
	*N* = 171	*N* = 3881	
Age, years, mean ± SD	72.6 ± 10.9	64.1 ± 10.8	<0.001
>65, *n* (%)	130 (76.0)	1863 (48.0)	<0.001
Gender, male, *n* (%)	127 (74.3)	2542 (65.5)	0.019
Smoking, *n* (%)	66 (38.6)	1498 (38.6)	1.000
Drinking, *n* (%)	53 (31.0)	796 (20.5)	0.001
Recent major bleeding^a^, *n* (%)	79 (46.2)	726 (18.7)	<0.001
History of gastrointestinal bleeding, *n* (%)	63 (36.8)	47 (1.2)	<0.001
History of peptic ulcers, *n* (%)	32 (18.7)	81 (2.1)	<0.001
Chronic kidney disease, *n* (%)	2 (1.2)	47 (1.2)	0.961
Chronic liver disease, *n* (%)	3 (1.8)	23 (0.6)	0.076
COPD, *n* (%)	6 (3.5)	70 (1.8)	0.115
Anemia^b^, *n* (%)	124 (72.5)	1948 (50.2)	<0.001
Diabetes, *n* (%)	60 (35.1)	846 (21.8)	<0.001
Hypertension, *n* (%)	125 (73.1)	2088 (53.8)	<0.001
Combined PPI, *n* (%)	42 (24.6)	1,634 (42.1)	<0.001
Combined oral anticoagulants, *n* (%)	62 (36.3)	159 (4.1)	<0.001
Combined NSAIDs^c^, *n* (%)	130 (76.0)	2860 (73.7)	0.498
Dual antiplatelet therapy, *n* (%)	62 (36.3)	732 (18.9)	<0.001
Causes of antiplatelet therapy			
Stroke or TIA, *n* (%)	39 (22.8)	818 (21.1)	0.588
ACS *n* (%)	103 (60.2)	2450 (63.1)	0.443
PAD, *n* (%)	7 (4.1)	204 (5.3)	0.503
CVD prevention, *n* (%)	22 (12.9)	409 (10.5)	0.334

GIB, gastrointestinal bleeding; SD, standard deviation; COPD, chronic obstructive pulmonary disease; PPI, proton pump inhibitors; NSAIDs, nonsteroidal anti-inflammatory drugs; TIA, transient ischemic attack; ACS, acute coronary syndrome; PAD, peripheral artery disease; CVD, cardiovascular/cerebrovascular disease.

^a^ Recent major bleeding: major bleeding <30 days before using antiplatelet drugs.

^b^ Anemia: defined as a hemoglobin concentration of <13 g/dl in males or <12 g/dl in females.

^c^ NSAIDs: included nonselective nonsteroidal anti-inflammatory drugs and celecoxib.

### Occurrence of Gastrointestinal Bleeding and Death

**FIGURE 2:**
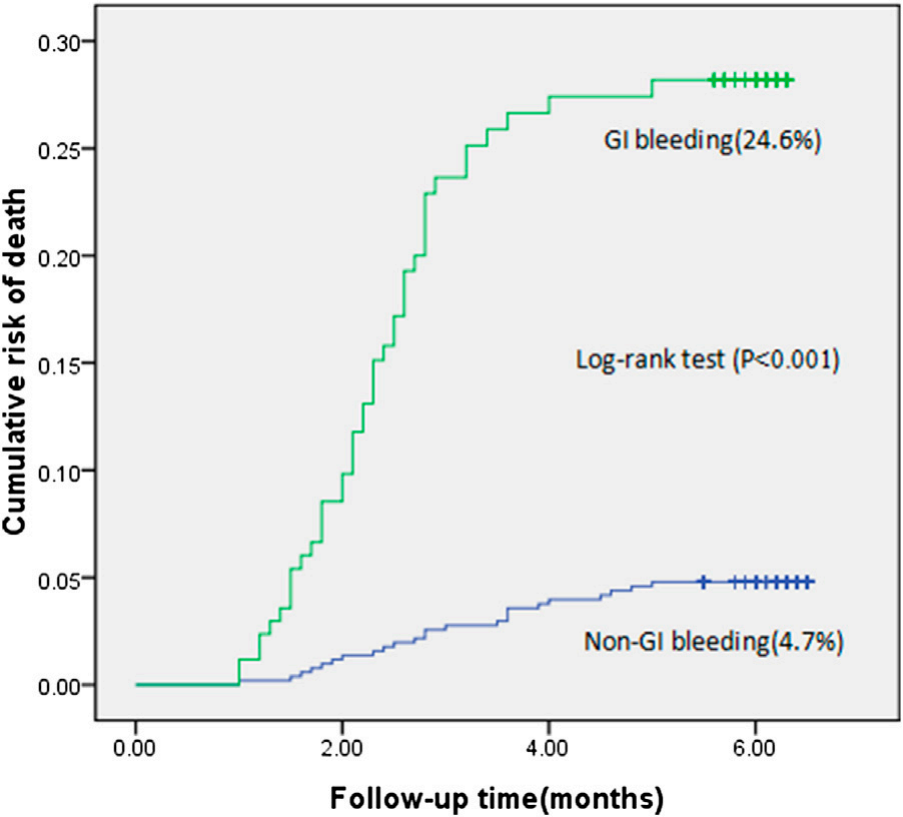
Cumulative risk of death in patients with gastrointestinal bleeding compared with patients without gastrointestinal bleeding (the logrank test, *p* < 0.001).

During the 6-month follow-up period, the incidence of acute gastrointestinal bleeding was 4.2% (171/4,052). The mortality rate in the bleeding group was 24.6% (42/171), and the mortality rate in the nonbleeding group was 4.7% (182/3,881). The mortality rate in the bleeding group was approximately five times that of the nonbleeding group (Figure 2).

### Risk Factors for Gastrointestinal Bleeding and Establishment and Evaluation of the Risk-Scoring Model

With a univariate analysis, factors related to acute gastrointestinal bleeding were an age of >65 years (*p* < 0.001), male sex (*p* = 0.019), drinking (*p* = 0.001), recent major bleeding (*p* < 0.001), a history of gastrointestinal bleeding (*p* < 0.001), a history of peptic ulcers (*p* < 0.001), chronic liver disease (*p* = 0.076), chronic obstructive pulmonary disease (*p* < 0.115), anemia (*p* < 0.001), diabetes (*p* < 0.001), hypertension (*p* < 0.001), combined use of proton pump inhibitors (*p* < 0.001), combined use of oral anticoagulants (*p* < 0.001), and dual antiplatelet therapy (*p* < 0.001).

With a multivariate analysis, the risk-scoring model of gastrointestinal bleeding was established using seven risk factors, including an age of >65 years, anemia, recent major bleeding, a history of gastrointestinal bleeding, no-proton pump inhibitors use, combined use of oral anticoagulants, and dual antiplatelet therapy (Table 2). The score was named the (ABC)_2_D score. According to the regression coefficient of the final model, an age of >65 years, anemia, recent major bleeding, a history of gastrointestinal bleeding, combined use of proton pump inhibitors, combined use of oral anticoagulants, and dual antiplatelet therapy were assigned 2.5, 1, 1.5, 6. −1.5, 3.5, and 1.5 points, respectively.

**TABLE 2:** Gastrointestinal bleeding risk with a univariate analysis.

Variables	OR (95% CI)	*p* value	Points
Age > 65 years	4.693 (2.411–9.132)	<0.001	2.5
Anemia^a^	1.864 (1.025–3.388)	0.041	1
Recent major bleeding^b^	2.788 (1.469–5.293)	0.002	1.5
History of gastrointestinal bleeding	39.204 (8.018–191.692)	<0.001	6
Combined PPI	0.446 (0.234–0.849)	0.014	−1.5
Combined oral anticoagulants	7.686 (2.841–20.789)	<0.001	3.5
Dual antiplatelet therapy^c^	2.410 (1.256–4.624)	0.008	1.5

OR, odds ratio; CI, confidence interval; PPI, proton pump inhibitors.

^a^ Anemia: defined as a hemoglobin concentration of <13 g/dl in males or <12 g/dl in females.

^b^ Recent major bleeding: major bleeding <30 days before using antiplatelet drugs.

^c^ Dual antiplatelet therapy: refers to the combined application of aspirin and clopidogrel.

The AUC for the (ABC)_2_D score was 0.857 (95% confidence interval (CI) 0.818–0.896; *p* < 0.001), which is higher than the CRUSADE score (0.693, 95% CI 0.637–0.749; *p* < 0.001; Table 3). The *p* value for the Hosmer–Lemeshow test was 0.324. The Hosmer–Lemeshow test indicated that the risk-scoring model had a good calibration capability (*p* > 0.05). Detailed score distribution of (ABC)_2_D score is shown in Table 4.

**TABLE 3:** Comparison of the predictive ability and performance of the (ABC)_2_D score and the CRUSADE score.

Score	Risk categories	Number (bleeding/no bleeding episode)	AUC (95% CI)	*p* value
(ABC)_2_D score			0.857 (0.818–0.896)	<0.001
≤2	Very low	8/69	—	—
2.5–5.5	Low	64/85	—	—
6–9	Moderate	44/12	—	—
9.5–12.5	High	29/5	—	—
≥13	Very high	26/0	—	—
CRUSADE score			0.693 (0.637–0.749)	<0.001
≤20	Very low	0/52	—	—
21–30	Low	37/38	—	—
31–40	Moderate	55/35	—	—
41–50	High	37/24	—	—
>50	Very high	42/22	—	—

AUC, area under the curve; CI, confidence interval.

**TABLE 4:** Detailed score distribution of (ABC)_2_D score.

Score	Total (*N* = 4,052)	GIB (*N* = 171)
−1.5	365	2
0	500	1
1	616	3
1.5	93	2
2.5	830	22
3.5	392	6
4	165	6
4.5	102	2
5	505	28
6	29	6
6.5	129	10
7	51	6
7.5	26	5
8	20	1
8.5	61	16
10	93	11
11	23	2
12	14	5
12.5	12	11
13	2	2
13.5	5	5
14.5	12	12
16	7	7

GIB, gastrointestinal bleeding.

## Discussion

We studied the incidence and mortality of gastrointestinal bleeding in patients taking oral antiplatelet drugs. Patients who experienced an episode of acute gastrointestinal bleeding had a significantly higher risk of subsequent death when compared with the nonbleeding group (24.6 vs. 4.7%, respectively; *p* < 0.001). Through univariate and multivariate analyses, it was found that an age of >65 years, anemia, recent major bleeding, a history of gastrointestinal bleeding, combined use of oral anticoagulants, and dual antiplatelet therapy increased the risk of gastrointestinal bleeding, while combined use of proton pump inhibitors reduced the risk of gastrointestinal bleeding. Therefore, it can help remind clinicians that proton pump inhibitors should be initiated for those with a high score or obvious risk factors. Furthermore, if the patient’s score is high, then the need for dual antiplatelet therapy or combined anticoagulant should be reviewed.

During the 6-month follow-up of patients taking antiplatelet drugs, the death rate of patients with gastrointestinal bleeding was 24.6%. Such a high mortality rate reflects the importance of developing an accurate and simple method to predict which patients are at a high risk of gastrointestinal bleeding. Therefore, we used seven factors to build a new gastrointestinal bleeding risk-scoring model, which has a better predictive power than the CRUSADE score. According to the AUC, this new risk-scoring model can distinguish the risk of gastrointestinal bleeding. We aim to use the (ABC)_2_D scoring system in clinical practice as it may be a useful method to reduce the risk of bleeding in patients taking oral antiplatelet drugs and to provide a certain reference for clinical antiplatelet therapy.

In our study, age and a history of gastrointestinal bleeding influenced gastrointestinal bleeding. This is consistent with the findings of Lin et al. (2013) who stated that age and a history of gastrointestinal bleeding are independent risk factors for gastrointestinal bleeding in patients taking aspirin and clopidogrel.

We also found that recent major bleeding events (odds ratio (OR) = 2.788; *p* = 0.002) and anemia (OR = 1.864; *p* = 0.041) increased the risk of gastrointestinal bleeding. Ruíz-Giménez et al., 2008 found that major bleeding events (e.g., gastrointestinal bleeding and intracranial hemorrhage) were related to recent major bleeding (OR = 2.7; *p* < 0.001) and anemia (OR = 2.1; *p* < 0.001), which is similar to our findings.

We also found that combined use of oral anticoagulants greatly increased the risk of gastrointestinal bleeding. Related studies have also shown that combined use of antiplatelet drugs and anticoagulant drugs is a risk factor for gastrointestinal bleeding. Like traditional anticoagulant drugs, new oral anticoagulant drugs may increase the risk of gastrointestinal bleeding (Abrignani et al., 2020).

In addition, related studies have shown that dual antiplatelet therapy has a higher bleeding risk than single antiplatelet therapy (Diener et al., 2004; Bhatt et al., 2006). Compared with aspirin alone, clopidogrel combined with aspirin causes a significantly higher bleeding rate (Yusuf et al., 2001). In two randomized controlled trials, the relative risk of gastrointestinal bleeding with dual antiplatelet therapy was almost twice that with single antiplatelet therapy (Yusuf et al., 2001; ACTIVE Investigators et al., 2009). We also found that patients taking aspirin in combination with clopidogrel had a higher risk of gastrointestinal bleeding (OR = 2.410; *p* = 0.008) compared with patients taking either aspirin or clopidogrel alone.

Finally, our study showed that the use of combined proton pump inhibitors reduced the risk of gastrointestinal bleeding by approximately half (OR = 0.446; *p* = 0.014), which closely reflects the findings of Hu et al., 2018, who stated that patients taking aspirin and clopidogrel used combined proton pump inhibitors, and the incidence of gastrointestinal bleeding decreased (OR = 0.58; *p* = 0.022). Although the combined use of proton pump inhibitors is not included in the CRUSADE (Subherwal et al., 2009) and S_2_TOP-BLEED scores (Hilkens et al., 2017), combined use of proton pump inhibitors is an important means of preventing gastrointestinal bleeding in the long term.

Our study is advantageous because of its relatively large sample size (*N* = 4,052). Our study presents the first risk-scoring model for predicting antiplatelet-induced gastrointestinal bleeding. Second, the statistical methods used in this study were rigorous. The AUC was used to evaluate the predictive ability of the model, while the Hosmer–Lemeshow test was used to assess the calibration ability of the model.

Our research also has some limitations. First, the study was conducted at two centers. Second, although (ABC)_2_D score is a better predictor than the alternatives, this would have to be proven on a new independent cohort in the future. Our next study will continue to collect more data from more centers, establish a new cohort, and further verify the scoring system. Third, our scoring tool has not yet been applied to the clinic on a large scale, so its versatility also requires further investigation in large-scale prospective multicenter research studies.

## Conclusion

We developed a new risk-scoring model based on seven factors (i.e., an age of >65 years, anemia, recent major bleeding, a history of gastrointestinal bleeding, no-proton pump inhibitors use, combined use of oral anticoagulants, and dual antiplatelet therapy) to evaluate the risk of acute gastrointestinal bleeding. The predictive ability of (ABC)_2_D is superior to that of the CRUSADE score. This new risk-scoring model may help to identify patients at a significant risk of gastrointestinal bleeding.

## Data Availability Statement

The raw data supporting the conclusions of this article will be made available by the authors, without undue reservation, to any qualified researcher.

## Ethics Statement

The studies involving human participants were reviewed and approved by the Fujian Medical University Union Hospital Ethics Committee. Written informed consent for participation was not required for this study in accordance with the national legislation and the institutional requirements.

## Author Contributions

JZ initiated the study. All the authors were involved in the literature search and data collection. ML and XZ performed the statistical analysis. ML drafted the first version of the manuscript. JZ and ML critically reviewed the manuscript and revised it.

## Funding

The work was supported by the National Natural Science Foundation of Fujian Province of China (grant no. 2018Y0037).

## Conflict of Interest

The authors declare that the research was conducted in the absence of any commercial or financial relationships that could be construed as a potential conflict of interest.
